# Dihydroquercetin in Weight Control: Systematic Review and Meta-Analysis of Preclinical Studies

**DOI:** 10.3390/ph18111675

**Published:** 2025-11-05

**Authors:** Roman P. Terekhov, Artem A. Svotin, Denis I. Pankov, Maria D. Korochkina, Elizaveta A. Krivosheeva, Elizaveta V. Krivozubova, Ketelina I. Bergel, Irina A. Selivanova

**Affiliations:** Nelyubin Institute of Pharmacy, Sechenov First Moscow State Medical University, Trubetskaya Str. 8/2, 119991 Moscow, Russia

**Keywords:** systematic review, meta-analysis, dihydroquercetin, taxifolin, flavonoid, obesity, weight control, preclinical studies, rats, mice

## Abstract

**Background**: Obesity is a global epidemic and a complex chronic disease affecting more than one billion patients, leading to severe health issues like diabetes, heart disease, and cancer. While lifestyle changes are the first-line treatment, they are often insufficient. Current medications may cause severe side effects, including muscle loss and vision problems. **Objectives**: This systematic review aims to generalize and evaluate data from preclinical studies on the effect of flavonoid dihydroquercetin (DHQ) on weight loss in experimental animals compared with placebo-treated animals. **Methods**: This systematic review was conducted in accordance with the PRISMA guidelines. The protocol was registered in the PROSPERO database in August 2025 (CRD420251129793). Risk of bias (RoB) was assessed by using SYRCLE’s tool. **Results**: In total, eight studies included in the systematic review involved 175 animals (14 treatment groups and 9 control groups). Calculation of correlations between the reported effect on weight change and initial weight showed a strong association between these rates (*R* −0.9883). The intensity of DHQ effect depended on the condition: There were strong negative correlations between DHQ dose and the observed effect in diabetes mellitus (*R* −0.9056), hepatic lipid dysmetabolism (*R* −0.9339), and hepatic fibrosis (*R* −0.9025) in mice and rats’ data together. **Conclusions**: Intake of DHQ in the course of one month and three months resulted in a decrease in animals’ weight by 5.24% ± 1.95% and 18.29% ± 1.96% (*p* < 0.0001), respectively. Taken together, our results suggest the rationality for further research of DHQ as an anorexigenic agent, focusing on the stereochemistry of this flavonoid and its bioavailability optimization.

## 1. Introduction

Obesity is a current challenge for modern healthcare systems. It is a complex chronic disease characterized by excessive accumulation of body fat that can negatively affect health [1]. Over the past 25 years, most particularly the last decade, momentum has been steadily building towards an acknowledgment that obesity is not merely a risk factor for illnesses such as type 2 diabetes, but it is a disease in its own right. In March 2021, the European Commission issued a brief in which obesity was defined as a “chronic relapsing disease, which in turn acts as a gateway to a range of other noncommunicable diseases” [2]. Obesity should no longer be regarded simply as an outward problem affecting certain individuals, but as an epidemic that threatens global well-being [3].

According to the World Health Organization, the total number of children, adolescents, and adults with obesity worldwide has exceeded one billion: 650 million adults, 340 million adolescents, and 39 million children [4].

Many countries are experiencing an increase in the number of people with obesity. In China, more than 400 million adults are overweight or obese. By 2030, the numbers of overweight and obese people in China aged 20–89 years are expected to reach 540 million and 150 million, respectively, which are 2.8 and 7.5 times higher compared to the prevalence in 2000 [5]. The latest European estimations show that approximately 53% of the adult population is overweight or obese. It was also found that in the countries of Eastern and Northern Europe, the obesity rate is higher than in other countries [6].

Excess body weight has a negative impact on health. The presence of excess adipose tissue leads to adverse health outcomes such as cardiovascular diseases [7], type 2 diabetes [8], musculoskeletal disorders [9], vision loss [10], and certain cancers like colorectal cancer [11]. Obesity can also affect sleep quality [12]. Furthermore, a strong link has been established between obesity and neurodegenerative disease. This issue is particularly urgent, as neurodegenerative diseases, including Alzheimer’s and Parkinson’s disease, currently affect over 60 million people worldwide and are the seventh leading cause of death globally [13]. All of this not only reduces the quality of life but also leads to premature death and significant disability [14]. Obesity adversely affects nearly all physiological functions of the body and comprises a significant public health threat [15].

Weight gain is associated with biochemical processes involving hormonal imbalances. A disruption in hormones such as cortisol, insulin, and leptin impairs metabolic regulation. Additionally, metabolic disorders and lifestyle factors contribute to the development of obesity [16].

Clinical guidelines emphasize lifestyle modification as the primary method to combat excess body weight: a healthy diet with calorie restriction and increased physical activity [17,18,19]. However, these methods have limitations. After stopping active efforts, body weight often returns or even exceeds the initial level [20]. Moreover, sustained success requires high motivation and constant self-monitoring, which can be challenging for many patients [21]. In such cases, a comprehensive medical approach is necessary, including behavioral interventions, pharmacotherapy, and bariatric surgery [22].

Bariatric surgery remains the most effective and long-lasting treatment method, demonstrating benefits not only for weight loss but also for cardiovascular and kidney health. It also helps reduce cancer incidence and obesity-related mortality. Long-term studies show that bariatric surgical procedures typically result in sustained weight loss of approximately 25% and a rapid, persistent reduction in obesity-related complications [23].

Nevertheless, the following drugs are registered for obesity treatment in the Russian Federation: orlistat, sibutramine, and liraglutide. It represents the current opportunities in drug selection for obesity treatment. Orlistat is a lipase inhibitor, and it has an important advantage: it acts only within the gastrointestinal tract without systemic effects. However, it has some side effects, including gallstone formation [24]. Sibutramine is a serotonin–norepinephrine reuptake inhibitor. Treatment with this drug requires strict medical supervision, as it is contraindicated in patients with uncontrolled arterial hypertension (blood pressure above 145/90 mm Hg), ischemic heart disease, decompensated chronic heart failure, arrhythmias, cerebrovascular diseases (stroke, transient ischemic attacks), occlusive peripheral artery diseases, age over 65 years, and severe liver or kidney impairments. It also has numerous side effects: nausea, loss of appetite, constipation, dry mouth, insomnia, headache, agitation, and sweating [25]. Liraglutide is an agonist of the glucagon-like peptide-1 (GLP-1) receptor. It regulates appetite by enhancing feelings of stomach fullness and satiety while reducing hunger and estimated food intake. One of the main advantages of this drug is that it can be considered a preferred option for patients with obesity and concomitant cardiovascular diseases. Typical side effects of this drug include nausea, vomiting, and diarrhea [26].

Globally, semaglutide, another GLP-1 receptor agonist, is actively used for obesity treatment. Available data indicate that rapid weight loss is accompanied by loss of skeletal muscle mass and function, which can lead to deteriorated functional status and metabolism, weight loss, impaired quality of life, and other adverse outcomes. Studies report that participants in clinical trials receiving incretin-mimetic drugs for obesity treatment lost 10% or more of their muscle mass over 68–72 weeks, which is equivalent to the muscle loss seen in 20 years of aging [27]. However, real-world clinical practice reported that semaglutide doubles the risk of developing neovascular age-related macular degeneration [28] and non-arteritic anterior ischemic optic neuropathy [29] in patients with diabetes.

So, the search for alternative medication in obesity is still a current objective for biomedical sciences, and the natural compounds seem to be promising objects of research due to their high safety profile. For example, vitamin B1 can influence glucose metabolism, and vitamin A shows a fat cell formation blocking effect in studies on animals [30]. Also, curcumin was used for obesity treatment. Studies on humans show that it reduces body weight, body fat, and waistline, lowers serum triglyceride levels, and improves cholesterol [31].

Anti-obesity effects have been found in certain polyphenol-rich foods like green tea, berries, citrus, coffee, cocoa, and ginger, which is why these products are often included in weight-loss diets [32]. In particular, flavonoids such as quercetin and kaempferol have anti-obesity effects. Weight loss is achieved by reducing calorie absorption, blocking fat accumulation, increasing calorie burning, and fighting underlying inflammation. Furthermore, these molecules have anti-oxidative properties, which also contribute to weight loss [33].

**Figure 1:**
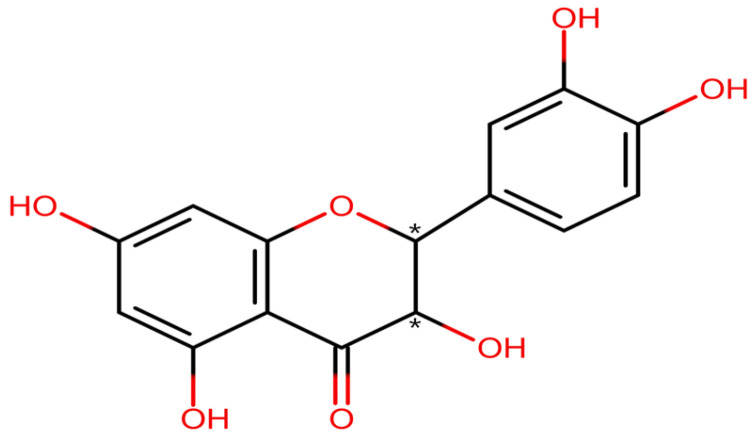
The structure of DHQ. Symbols * show the position of stereocenters.

Among other natural compounds, we will focus on the major flavonoid component of larch wood—dihydroquercetin (DHQ), which is also known as taxifolin (Figure 1). This natural polyphenol is widespread as a plant-based raw medicinal material [34,35,36] and is also frequently used in food production [37,38,39]. In addition, DHQ is found in some edibles [40,41,42]. It is important to mention some pharmaceutically valuable characteristics of the taxifolin molecule: The molecular weight is 304.25 g/mol, LogP is 1.57 [43], and the topological polar surface area is 127 Å^2^ [44]. Based on these data, it can be said that DHQ is a small polar molecule with low water solubility. Also, a large number of hydroxyl groups acting as H-bond donors determines its ability to bond with different biological targets in active sites [45].

It has recently been observed that DHQ has a beneficial effect on weight loss [46]. Furthermore, there are a lot of studies in bibliographical databases that record weight dynamics on the background of consuming this flavonoid in people [47,48], rats and mice [49,50,51], and other species [52,53,54]. But the data on this topic has not been systematized yet. Therefore, research into the effect of DHQ on weight loss is extremely relevant.

This systematic review aims to generalize and evaluate data from preclinical studies on the effect of DHQ on weight loss in experimental animals compared with placebo-treated animals.

## 2. Materials and Methods

### 2.1. Protocol

This systematic review was conducted in accordance with the PRISMA guidelines [55], which regulated the search strategy, study selection, data extraction, and analysis. The protocol was registered in the International Prospective Register of Systematic Reviews (PROSPERO) database in August 2025 (CRD420251129793) [56]. Patients or public partners were not involved in the design, conduct, or interpretation of this systematic review.

### 2.2. Search Strategy

The relevant literature was identified through searches of the PubMed, Google Scholar, and eLibrary databases, with no restrictions applied to the publication date. The search strategy utilized the following key terms: “taxifolin”, “dihydroquercetin”, “weight”, “fat”, “obesity”, and “body mass”. An example of the search strategy applied to the scientific databases is provided in English: (taxifolin OR dihydroquercetin) AND (weight OR fat OR obesity OR “body mass”). In Russian, the equal terms were used.

Two researchers (M.D.K. and E.A.K.) independently and simultaneously performed an initial search and screening of articles by reading their titles and abstracts to form the reference list. In case of disagreements, they were resolved by another author (R.P.T.). The overall inclusion and exclusion criteria that were used during screening are presented in Table 1.

### 2.3. Data Extraction and Synthesis

Data extraction was performed independently by two researchers (E.A.K. and K.I.B.) who collected mean and standard deviations (SDs) of body weight data (specified or converted in grams) at week 4 or week 12 after the start of DHQ intake from the main text, tables, figures, and Supplementary Materials of the selected articles. In cases of discrepancies, the third researcher served as a referee. The results of the extraction were compiled and summarized in a Google Sheets spreadsheet. The same researchers then independently screened the publications for eligibility based on the predefined inclusion and exclusion criteria. Disagreements were resolved through discussion between the reviewers. If consensus was not reached, a third author (R.P.T.) was consulted.

Both qualitative and quantitative content analyses were applied. Data synthesis and statistical analysis were performed by M.D.K., E.A.K., E.V.K., and K.I.B. The results are presented as a narrative summary, supported by tables and figures.

### 2.4. The Key Data

The primary effect measure for this systematic review and meta-analysis was the mean difference in body weight change between the control and experimental groups, or the effect size (*ES*). This measure was calculated using the following formula:

$$ES=\left(\frac{\Delta m_{e}}{m_{e,i}}-\frac{\Delta m_{c}}{m_{c,i}}\right)\times 100\%,$$

where Δ*m_e_*—the mean difference between the initial and the final body weight of the experimental group, *m_e_*_,*i*_—the initial body weight of the experimental group, Δ*m_c_*—the mean difference between the initial and the final body weight of the control group, and *m_c_*_,*i*_—the initial body weight of the control group.

### 2.5. The Forest Plot Construction

The *ES* and its 95% CI for each study were calculated for inclusion in the forest plot. The methodology for variance estimation was as follows.

For each study, an *F*-test was performed on the sample variances of the experimental and control groups to determine the appropriate method for calculating the standard error of the effect size (*SE_ES_*). Based on the outcome of this test (i.e., whether the variances could be considered equal or unequal), the corresponding formula for the *SE* of the difference between two independent means was applied.

For studies that provided *SE* values for the initial and final measurements, the combined *SE* for the mean difference within each group was first calculated. Subsequently, the *SE* for the mean difference was computed by the following formula:

$${SE}_{MD}=\sqrt{{{(SE}_{e})}^{2}+{{(SE}_{c})}^{2}},$$

where *SE_e_*—the standard error of the mean difference between the initial and the final body weight of the experimental group, and *SE_c_*—the standard error of the mean difference between the initial and the final body weight of the control group.

The weight of each study (*w*) was calculated as the number of individuals in the experimental and control groups of the study divided by the total number of individuals in the study.

The position and width of the diamond were calculated using the following formula:

$$WMD=\frac{\Sigma ({ES}_{i}\;\times {\;w}_{i})}{\Sigma w},$$

where *WMD*—the weighted mean difference, *ES_i_*—the effect of *i*-th study, *w_i_*—the weight of *i*-th study, and Δ*w*—the overall weight.

$$CI=WMD\pm 1.96\times \sqrt{\frac{1}{\Sigma w}},$$

where *WMD*—the weighted mean difference, Σ*w*—the overall weight.

*p*-values were calculated using *z*-statistics. For *WMD*, the effect size of each study included in the data graph was taken into account during the calculation of the *p*-value.

Next, the data heterogeneity was assessed using the *Q*-test, resulting in the values of heterogeneity (*Q*) and the heterogeneity index (*I*^2^) of each plot.

### 2.6. The Funnel Plot Construction

All groups included in the systematic review were applied to the funnel plot, but only the values recorded on week 4 were taken. The *y*-axis is a group size, and the *x*-axis is *ES* to DHQ dosage (mg/kg animal weight) ratio (Δ, %/mg/kg). Subsequently, the *ES*/dosage ratio was calculated for each included group.

This data was used to make a funnel plot. Then, based on the obtained values, the placement of the middle of the average was found.

### 2.7. Assessment of Risk of Bias

The risk of bias assessment was performed independently by two researchers (M.D.K. and E.V.K.) using SYRCLE’s RoB tool [57]. The results were then summarized. In case of discrepancies, a third researcher (A.A.S.) served as a referee.

## 3. Results

### 3.1. General Outlook on the Scientific Landscape

**Figure 2:**
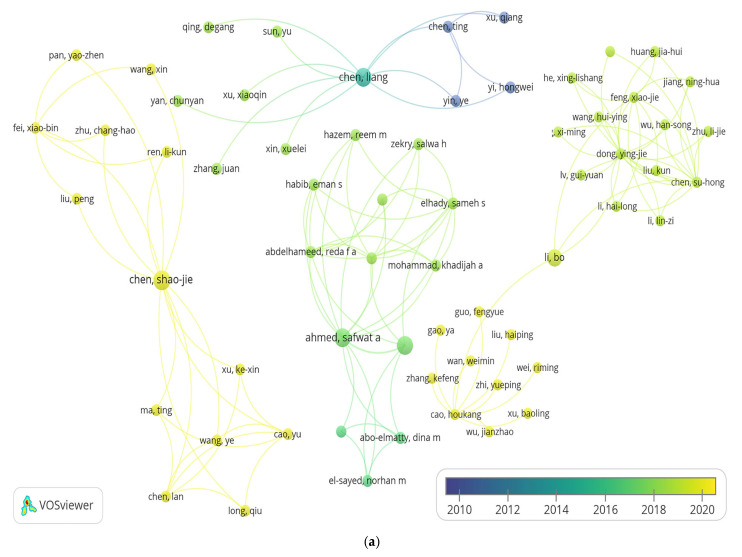

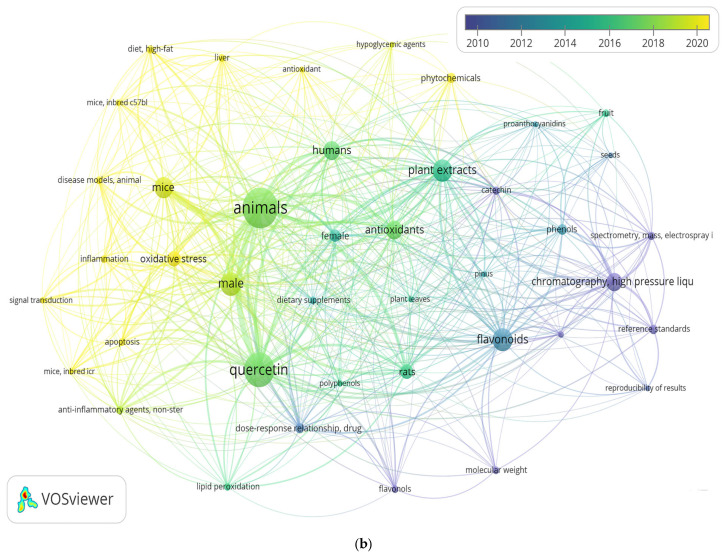
Bibliometric network of terms based on data from PubMed: (**a**) co-authorship analysis and (**b**) co-occurrence analysis. Created by VOSviewer version 1.6.20 [58,59].

To assess the relevance of the study, data published on PubMed were collected. These data were used to build a bibliometric network based on co-authorship (Figure 2a) and on keywords co-occurring with the terms “taxifolin”, “dihydroquercetin”, “obesity”, and “body weight” in articles (Figure 2b). The size of bubbles corresponds to the frequency of term mentions. The pseudo-color scale reflects the novelty of articles from years earlier than 2010 (dark-blue) up to 2025 (yellow).

Four examples of the connection between author teams are shown. Liang Chen’s team from the State Key Laboratory of Pharmaceutical Biotechnology in the School of Life Sciences of Nanjing University (Nanjing, China) studies plant chemical compounds, including flavonoids, using physicochemical methods such as chromatography and performing experiments on mice [60,61]. The team of Shao-Jie Chen from Guizhou Medical University in China is focused on the study of apoptosis in pancreatic and melanoma cancer cells [62,63]. Safwat A. Ahmed from the Department of Pharmacognosy, Faculty of Pharmacy (Suez Canal University in Egypt) and his team study effects of plant extractions on diseases such as hepatic and renal toxicities [64], search for new antitumor drugs, and examine the effect of plant compounds on obesity in rats [65]. Bo Li from Zhejiang University of Technology (Hangzhou, China) connects two teams: one that studies the treatment of alcoholic liver disease [66,67] and the other that studies therapeutic effects and mechanisms of plant compounds [68,69]. It does not go unnoticed that Liang Chen’s team’s work dates back a decade, unlike Shao-Jie Chen’s and Bo Li’s teams, whose research is more recent, as indicated by the yellow color of the bubbles.

The terms “animals”, “quercetin”, “flavonoids”, “plant extracts”, “mice”, and “male” are the most frequently used, which is indicated by their bubble size. There is a shift in interest from physicochemical methods such as spectrometry and chromatography [70,71], showed as dark-blue bubbles, to studies on animals (“disease models, animal”, and “mice, inbred ICR”) in different conditions, e.g., “inflammation”, “apoptosis”, and “hypoglycemic agents” [51,62], presented as yellow bubbles.

Thus, DHQ is currently at the stage of active preclinical research, confirming the need for data systematization, which is the purpose of this review.

### 3.2. Study Selection

**Figure 3:**
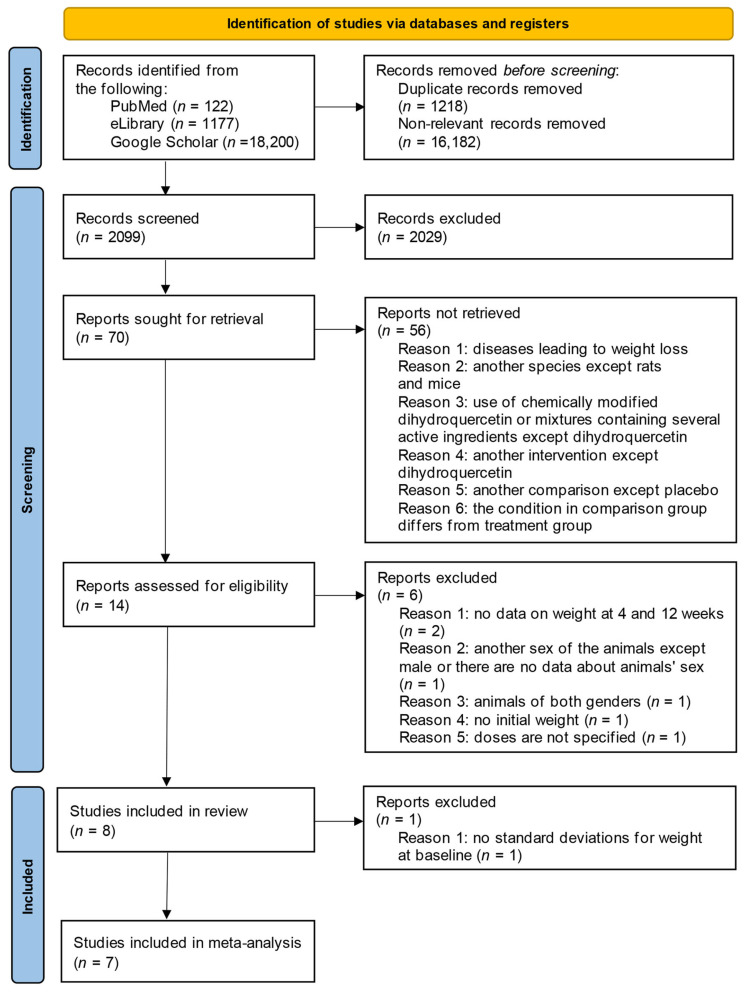
PRISMA flow diagram.

The collection and selection process is illustrated in the Preferred Reporting Items for Systematic reviews and Meta-Analyses (PRISMA) flow diagram (Figure 3). A total of 19,499 articles were identified through the search strategies of PubMed, eLibrary, and GoogleScholar.

Before the screening, 1218 duplicates and 16,182 non-relevant records were removed. After the first screening, 2029 articles were excluded because they did not meet the inclusion criteria based on their titles and abstracts. In the subsequent review, 56 articles were eliminated for the following reasons: the diseases leading to weight loss (colitis [72,73], cancer [62], burns [27,74], myocardial or kidney injury [75], pulmonary fibrosis [76,77], hypoxia [78], and hepatitis [79]) the research was conducted on animals other than mice or rats [80,81], the use of chemically modified DHQ or mixtures containing several active ingredients except DHQ [82,83], another intervention except DHQ intake [84], and another comparison except placebo or condition in comparison group differs from treatment group [81]. After reviewing the full texts, an additional six articles were excluded for the following reasons: there was no data on weight at the end of week 4 and week 12 (*n* = 2) [49,50], no information was included about gender and weight (*n* = 1) [85], animals of both genders were included (*n* = 1) [49], there was no data on initial weight (*n* = 1) [86], and doses were not specified (*n* = 1) [51]. Consequently, eight articles passed through all stages of selection and were included in the systematic review. However, only seven articles were appropriate for meta-analysis due to the absence of SD for weight at the baseline in one of the articles included in the review [87].

### 3.3. Study Characteristics

The studies were conducted across diverse countries, such as Russia, China, Japan, and Egypt, with sample sizes varying from 6 to 10 animals. Of all included studies (Table 2), 107 mice (eight treatment groups and six control groups) and 31 rats (two treatment groups and two control groups) were included in the meta-analysis. As for the systematic review, 144 mice (12 treatment groups and 7 control groups) and 31 rats (2 treatment groups and 2 control groups) were involved. Included studies encompassed diabetes mellitus types 1 and 2 [88,89,90], hepatic fibrosis [90,91], gonadal dysfunction [92], hepatic lipid dysmetabolism [87,93], obesity [94], and healthy state models [93].

Mean initial weight and age were 31.2 ± 11.8 g and 10.0 ± 6.0 weeks for mice and 227.5 ± 74.3 g and 8.0 weeks for rats, respectively. Most studies incorporated mice with an initial weight ranging from 17.3 g to 28.6 g and an initial age varying from 4 to 8 weeks, but in one study, the initial weight of mice was 40.8–48.3 g and the initial age was 17–19 weeks [93]. In this way, there was no significant difference within groups of the same species. Treatment and control groups can be characterized as homogeneous in all included studies.

### 3.4. Narrative Synthesis

Overall, six of eight studies reported on the weight loss in DHQ treatment groups compared with the control groups (Table S1).

The most pronounced weight loss was observed with DHQ suspension in saline solution in the streptozotocin-induced diabetes mellitus rats at a dose of 50 mg/kg. At the end of week 4, body weight in the DHQ group decreased to 25.41% compared to only an 8.64% reduction in the control group [88]. Another notable result was observed for DHQ in the dose of 80 mg/kg, administered orally in mice with hepatic fibrosis and diabetes mellitus, which resulted in a −10% of weight in the treatment group, compared with the control group. Slight increases in body weights in DHQ groups were observed in rats with gonadal dysfunction (+7.02%, 50.0 mg/kg dose) and obesity (+5.71% and +4.12% for doses 41.2 and 73.6 mg/kg, respectively).

Several studies have reported that DHQ contributes to reducing blood glucose and triglyceride levels compared to the control group. The greatest reduction in glucose was observed at week 12 of treatment with oral administration of DHQ at a dose of 80 mg/kg, where the difference between the control and experimental groups reached 37.93% [90]. A similar trend is observed for triglyceride levels. The maximum reduction in triglycerides compared to the control group was observed with oral administration of DHQ at 25 mg/kg, yielding a 42.3% difference between the experimental and control groups [93].

It was also noted that longer treatment durations resulted in more pronounced effects, as evidenced by a study showing a 10% difference by week 4, which increased to 50% by week 12 [90].

**Figure 4:**
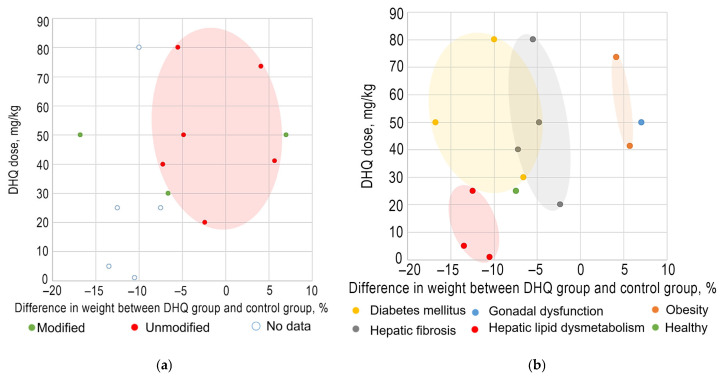
Subgroup analysis of the efficacy of body weight loss depending on (**a**) the modification of DHQ solubility and (**b**) the condition of animals.

In all included studies, the administration of DHQ was administered as a single daily dose, either orally or by intragastric injection, at various dosages. The DHQ doses were based on the individual weights of animals and varied from 1 to 80 mg/kg. The duration of treatment ranged from 4 to 12 weeks. DHQ was administered in the form of suspension in water [94] and oil [87] liquid mediums. To a large extent, the results of the experiments depended on the chosen solvent. According to the data presented in Table S1, better outcomes were achieved when DHQ was suspended in saline solution: The efficacy of such formulation was 1.65 times higher compared to corn oil [87,88,89]. In some cases, the solubility of DHQ was altered by phase modification [88] or the addition of solubilizers in the solution, such as methyl cellulose [92] and sodium carboxymethyl cellulose [89]. Notably, in the case of weight loss, the application of formulations with enhanced DHQ solubility was associated with higher biological effects at lower doses (Figure 4a). The route of DHQ administration also influenced the weight loss. Intragastric injections, in some cases, produced a smaller difference between the DHQ and control groups compared to oral administration. At identical DHQ dosages (80 mg/kg), oral administration resulted in a 1.82-fold greater weight loss than intragastric administration [90,91].

Weak positive correlations for DHQ dose and difference in body weight change between the treatment and control groups (%) at the end of week 4 were observed: the correlation coefficients were 0.4164 and 0.2508 for the mice subgroup and for both species, respectively. However, the analysis of subgroups, based on the conditions, showed reverse results. There were strong negative correlations between DHQ dose and the observed effect on diabetes mellitus (*R* −0.9056), hepatic lipid dysmetabolism (*R* −0.9339), and hepatic fibrosis (*R* −0.9025) in mice and rats’ data together. Furthermore, the intensity of the biological effect depended on the condition (Figure 4b). The differences between DHQ groups and control groups in the body weight changes were clearer in diabetes mellitus and hepatic lipid dysmetabolism than in hepatic fibrosis, obesity, gonadal dysfunction, or healthy animals. Compared to untreated animals, the body weight of treated ones decreased by almost half. Additionally, research shows that the reducing blood glucose effect of DHQ becomes more pronounced with the increase in dosage. For example, with oral administration at a dose of 50 mg/kg, blood glucose levels at week 4 were 8.55% lower compared to the control group, whereas at 80 mg/kg, the difference at week 4 reached 21.74%. The decrease in triglyceride levels is also dose-dependent: A higher dose results in a more pronounced effect. For instance, administration of DHQ at 1 mg/kg resulted in a 24.5% reduction compared to the untreated group, at 5 mg/kg the reduction was 35.6%, and at 25 mg/kg it reached 42.3% [93].

**Figure 5:**
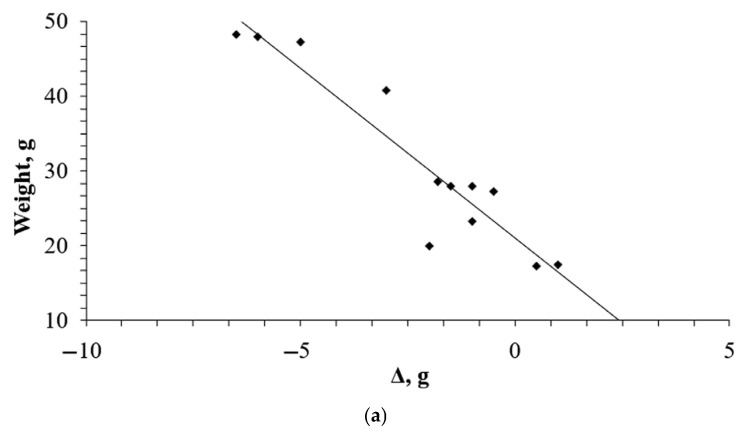

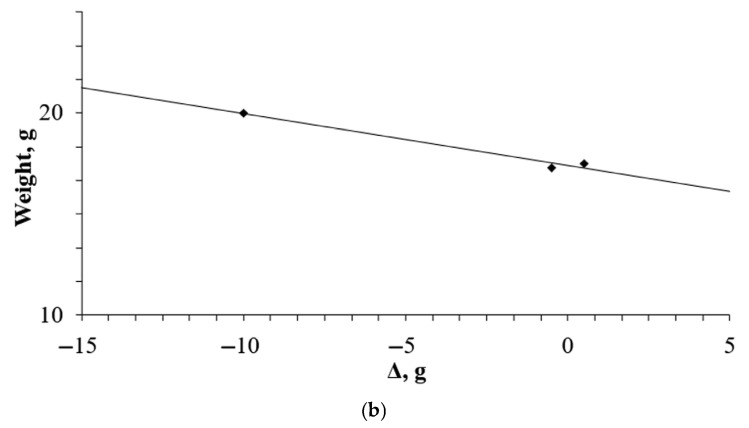
Correlations between the reported effect on weight changes and initial weight for mice (**a**) for week 4 and (**b**) for week 12.

Calculation of correlations between the reported effect on weight change and initial weight (Figure 5) showed a strong association between these rates for both weeks 4 (*R* −0.9367) and 12 (*R* −0.9883) of the study.

Also, there is a strong correlation between the observed effect on weight change and initial age (*R* −0.8871 for mice for the week 4 and *R* −0.9963 for mice for the week 12). Within the same animal species, weight and age have direct relations, so correlations of these rates with the observed effect on weight change are almost similar.

### 3.5. Quantitative Analysis

The meta-analysis included eight studies that reported the necessary data for eleven subgroups, such as SD or SE. Based on the observed outcomes and the species analyzed, the information was divided into four forest plots. Data on the change in rat body weight at week 12 were not available in these studies. Therefore, the plot illustrating the overall results for body weight change at week 12 and the plot displaying the data for mouse body weight at week 12 were combined into one plot.

**Figure 6:**
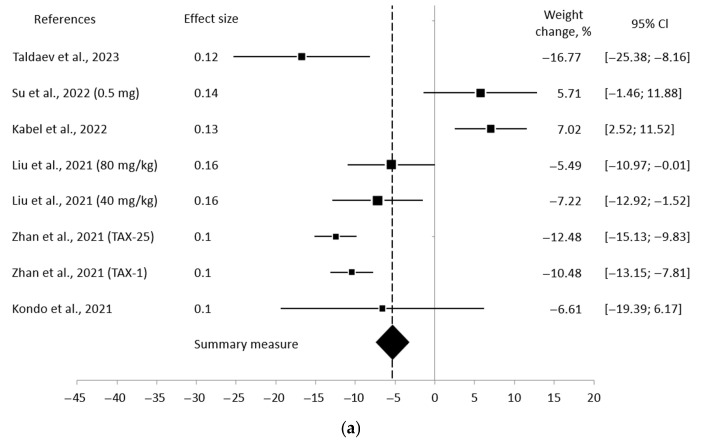

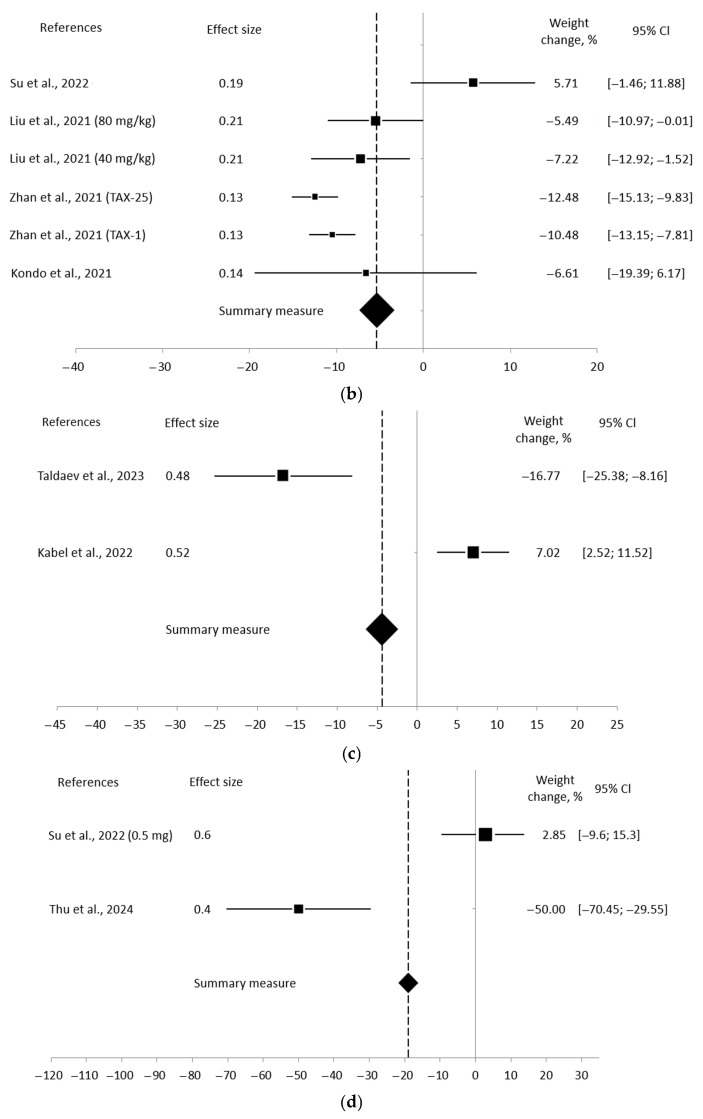
Forest plots showing the mean differences in body weight change between experimental and control groups across various species at different time points: (**a**) mice and rats at week 4 [88,89,91,92,93,94]; (**b**) mice at week 4 [89,91,93,94]; (**c**) rats at week 4 [88,92]; and (**d**) mice at week 12 [90,94].

Figure 6a shows that the mean differences ranged from −16.77% to 7.02%. In this scenario, the weighted mean difference was −5.24% (95% CI: −7.19 to −3.29) with *p* < 0.0001 and high heterogeneity (*Q* 61.09, *I*^2^ 86.90%). By contrast, one article, despite a negative average outcome, reported that some animals in the experimental group experienced a greater increase or a smaller decrease in body weight compared to the controls [89]. On the contrary, another study demonstrated the opposite result [94]. It presented the positive result, but only in the context of the mean difference: 5.71% (95% CI: −1.46 to 11.88). A positive mean difference of 7.02% (95% CI: 2.52 to 11.52) was observed by Kabel et al., which significantly differed from the average outcome [92].

Further, the plot of data on body weight change obtained from mice at week 4 (Figure 6b) showed a similar weighted mean difference: −5.44% (95% CI: −7.39 to −3.49), *p* < 0.0001. Also, the results had high heterogeneity among themselves (*Q* 34.22, *I*^2^ 82.47%). The mean differences ranged from −12.48% to 5.71%. It is worth noting that the trend observed in the aforementioned studies was also maintained in this plot.

Figure 6c demonstrates that the mean differences were −16.77% and 7.02%. The weighted mean difference was −4.40% (95% CI: −6.36 to −2.44) with *p* 0.000032 and a high heterogeneity (*Q* 141.26, *I*^2^ 99.29%). Referring to this plot, the above-mentioned trend observed in the article by Kabel et al. was also preserved [92]. Additionally, these results are not statistically different from the results of body weight change between experimental and control groups of mice (CI overlap).

The plot of data on body weight change obtained from mice at week 12 (Figure 6d) showed a mean difference of −18.29% (95% CI: −20.25 to −16.33), *p* < 0.0001, which was notably lower than the mean results at week 4. On the other hand, one of the included studies showed a mean difference of 2.85% (95% CI: −9.60 to 15.30). This indicates that the body weight of some mice in the experimental group was either increased to a greater extent or decreased to a lesser extent than in the control group. It is important to note that the *p*-value of this study was 0.33 [94]. It means that the results of this study were insignificant. They were also considered not significant, since the *p*-value for one of the studies was 0.33, and the *p*-value for the average outcome was > 0.05. Also, the results between themselves had high heterogeneity (*Q* 670.35, *I*^2^ 99.85%), which must necessarily be considered in the future interpretation of this forest plot.

Therefore, examining the results using the forest plots showed that most estimates evidenced a pronounced weight loss effect due to DHQ administration.

### 3.6. Risk of Bias Assessment

**Figure 7:**
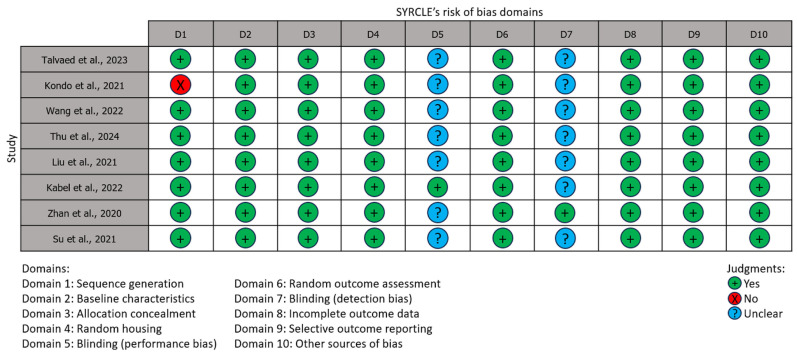
Risk of bias. Source [87,88,89,90,91,92,93,94].

Risk of bias was assessed for eight studies using SYRCLE’s RoB tool for animal studies (Figure 7). All studies exhibited performance or detection bias, with six of them being 20% “Unclear” because of both. One study was 10% “Unclear” due to the absence of information on blinding caregivers and researchers [92], and another one due to the lack of information on blinding of the outcome assessors [94]. Only one article was rated as “No” in the sequence generation domain, indicating selection bias [89]. This resulted in a 10% bias rate and a 20% unclear risk of bias in this study.

**Figure 8:**
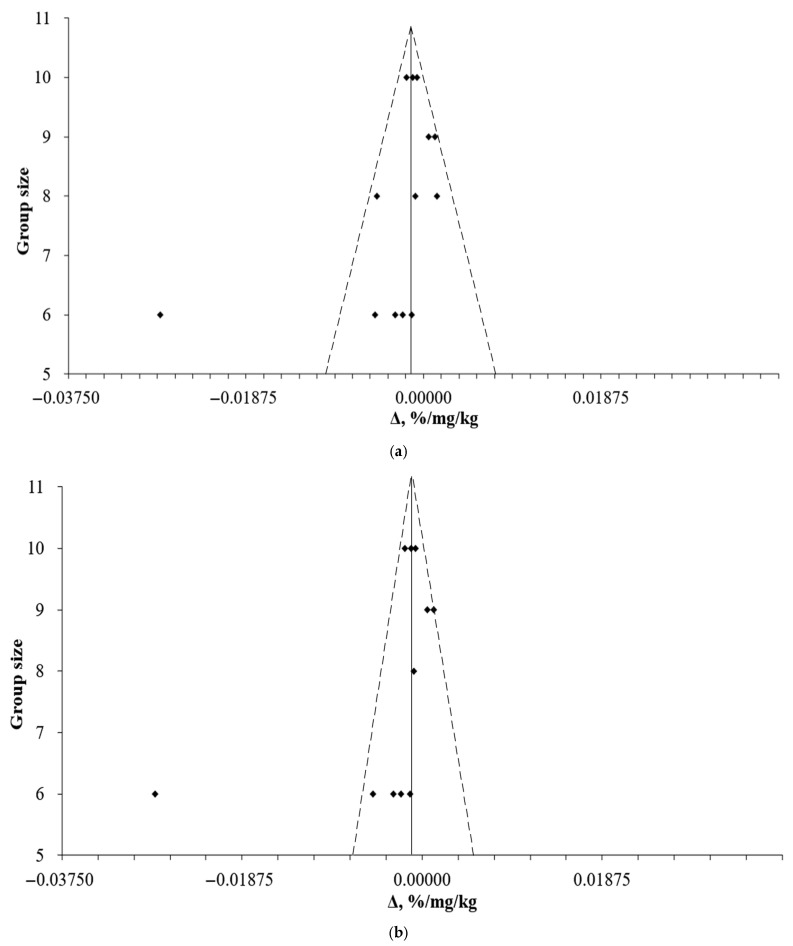
Funnel plots, illustrating the link between group size and observed effect on weight change (**a**) for both animal species and (**b**) for mice.

The funnel plots (Figure 8) showed that the observed effect on weight change in studies with bigger sample sizes tended to −0.0019%/mg/kg for both animal species and to −0.0015%/mg/kg for the mice subgroup. Furthermore, there is more data shifted towards weight loss with the use of DHQ.

## 4. Discussion

### 4.1. Relevance of the Systematic Review

An initial objective of this systematic review was to identify the trend of weight loss in rats and mice under the influence of DHQ. This flavonoid has a long history of medical application, and its wide range of biological effects makes it suitable for various therapeutic approaches. The analysis of the scientific landscape highlights the relevance of the present study, as evidenced by the significant number of research groups worldwide and the corresponding volume of publications in bibliographic databases. This interest in DHQ for weight management is driven by several factors. Firstly, DHQ exhibits a high safety profile, which is particularly appealing in light of the emerging safety concerns associated with other weight loss interventions, such as semaglutide [95,96]. Secondly, the bibliometric network analysis of co-authorship reveals that the leading research teams in this field are primarily based in countries of the Global South, where herbal medicinal products often serve as frontline therapies [97]. Finally, a comprehensive review is timely because the scientific community has largely progressed beyond basic physical and chemical characterization of DHQ, and, as shown on the bibliometric network of terms, is now increasingly focused on preclinical studies to evaluate its efficacy and mechanisms of action in vivo, and these data require consolidation and systematization.

### 4.2. Review Design Rationale

The weight loss potential owing to DHQ was investigated in mice and rats frequently, as their metabolism and mechanisms of weight gain are similar to those of humans, and they serve as relevant models for studying processes occurring in the human body due to their high genetic similarity [98]. In the current review, exclusively male animals were included to eliminate the influence of female sex hormones [99], which can affect the results. We also excluded studies involving animals with conditions such as colitis [72,73], cancer [62], burns [74,100], myocardial or kidney injury [75], pulmonary fibrosis [76,77], hypoxia [78], and hepatitis [79] from the analysis, as these pathologies can independently induce weight loss and lead to inaccurate conclusions regarding the effects of DHQ. For the same reason, we included only articles in which pure DHQ was used as the active substance, in order to avoid any distortion of the results. Due to differences in the age and body weight of animals in the reviewed studies, we presented all data as % to standardize the results, calculated as the difference between the treatment group and the control group. As the authors of this research are fluent in English and Russian, the papers in other languages were excluded from the following analysis to avoid misunderstandings that are associated with machine translation of scientific texts. Most studies involve growing mice [87,89,90,92,94] and rats [93] that continued gaining weight. So, we presented the data as a comparison between treatment and control groups to minimize variability.

### 4.3. Major Outcomes

Applying all the limitations mentioned above, a sample size of over 175 animals was obtained, which seems acceptable for the biological effect synthesis. Included studies took place in diverse regions, which increases the generalization of the results. No critical deviations from the weight baseline among animals of the same species were observed. Contributions of treatment groups from included studies are roughly comparable (from 0.10 to 0.16). Nonetheless, reported results are highly heterogeneous due to differences in pathological states.

The results of the risk of bias assessment suggest that the analyzed articles generally meet the standards of good preclinical study practice, and these results are reliable. However, domains 5 and 7 being “Unclear” in almost every article presume that giving detailed information about blinding is not a strict rule in preclinical studies, in contrast to such demand in clinical trials. Therefore, it seems that the fact of blinding itself is enough, making uncertainty about these biases negligible. Nevertheless, one article was biased in the sequence generation domain due to the absence of information on randomization. This may be problematic, as randomization is aimed at ensuring comparability of the results between treatment and control groups. Unlike Cochrane’s, SYRCLE’s RoB tool does not have the “Overall” column. SYRCLE’s authors do not recommend calculating a summary score for each individual study due to the difficulty of justifying the weights assigned [57].

Analysis of the results using forest plots revealed that most rates indicated a significant body weight loss effect following DHQ administration in both mice and rats. At the same time, this flavonoid contributed to the reduction in blood glucose and triglyceride levels. This suggests that DHQ shares similar biological targets with medications that decrease body weight and normalize the blood chemistry value.

The assessment of publication bias is important because it affects the validity of the conclusions drawn from a meta-analysis. The presented funnel plots are almost symmetrical, indicating that there is no significant publication bias. However, when attempting to replicate the data on animals with the concentration that showed the best results in weight loss, the real average result may be more modest, due to the lack of articles that were performed on small samples and reported the absence of difference between treatment and control groups. It is interesting to notice that the tendency to gain body weight with DHQ administration is lower when the initial weight is higher. For example, the difference between the initial and final body weight of the experimental group at week 4 in the one of included articles was a gain of 5.0 g [87], while in another one it was a gain of 6.0 g [90], given that the initial weight in the first study was 23.5 g and in the next it was 20.0 g. However, when comparing the results obtained by Porter et al. [101] and Deng et al. [102], this dependence was not observed (the differences at week 4 were −2.3 g and −2.6 g, with initial body weights of 57.6 g and 24.4 g, respectively).

### 4.4. Hypotheses on the DHQ Pharmacodynamics in Obesity

The first probable mechanism involves the effect of DHQ on insulin receptors and insulin signaling pathways. For example, Gao et al. describe the activation of the PI3K/AKT signaling pathway in the kidney, contributing to the maintenance of renal glucose homeostasis through DHQ administration: PI3K and p-GSK3β levels increase, while p-GS, SGLT-2, and GLUT-2 levels decrease [50]. Additionally, the flavonoid inhibits the activation of the RAAS, which also contributes to glucose reduction. An interesting finding is that GLP-1 receptor agonists, such as semaglutide, liraglutide, and tirzepatide, exert similar effects on these biological targets [103].

The second mechanism is related to lipogenesis enzymes. Zhang et al. illustrate this point clearly [104]. It is reported that DHQ reduces lipid synthesis and increases its oxidation through activation of the LKB1/AMPK pathway, inhibition of SREBP1, promotion of ACC phosphorylation, and upregulation of SIRT1. Notably, the flavonoid restores insulin sensitivity by suppressing inflammation via NLRP3, caspase-1, and IL-1β, which also impacts glucose levels. Surprisingly, the aforementioned medications have a similar effect [105].

Finally, effects on adipose tissue and mitochondrial function contribute to body weight loss. The influence on cellular “power plants” is exemplified by Khadrawy et al., who consider the Bcl2/Bax/Caspase-3 signaling pathway [106]. A decrease in macrophage-produced IL-1β and TNF-α is demonstrated [107]. Among medications with mechanisms of action similar to DHQ, metformin can be highlighted, as it improves mitochondrial function and moderately reduces body weight [108]. Furthermore, GLP-1 receptor agonists indirectly affect this organelle and also reduce inflammation [28].

**Figure 9:**
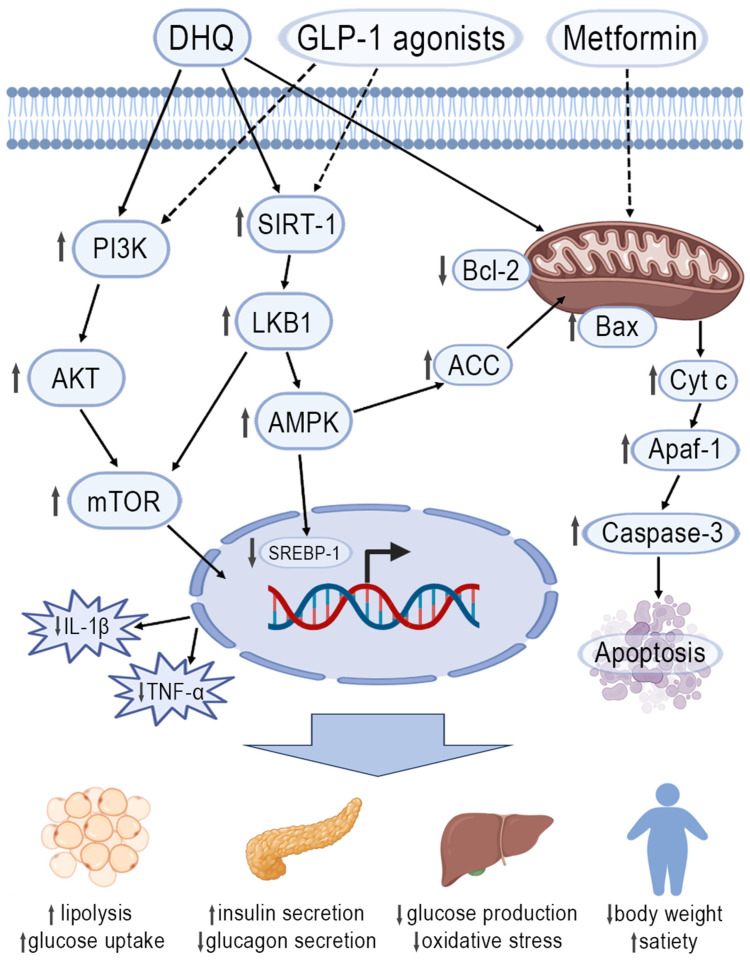
Sum of possible molecular mechanisms of DHQ anorexigenic effect.

These hypotheses on the DHQ pharmacodynamics are summarized in Figure 9.

As DHQ and GLP-1 receptor agonists seem to have similar biological targets, it may be interesting to compare their anorexigenic effects in preclinical studies. In a study on Swiss TO mice, it was reported that the mean difference in body weight change at week 4 between the experimental group administered liraglutide and the control group was −7.43% ± 10.34% [101]. In a similar study on KK-Ay/Ta mice, this number was −6.61% ± 9.36% [89]. This is a highly remarkable result. In a clinical study of liraglutide at a concentration of 3 mg [109], the mean difference in body weight change at week 4 between the experimental and control groups was −1.82% (for patients with a height of 1.70 m and body mass index of 30 kg/m^2^). This result is consistent with data from the summarized data of DHQ studies in mice: The weight loss was −5.44% ± 1.95% at the end of week 4. It is important to highlight that the liraglutide treatment effect is clinically significant, as the experimental group achieved rapid weight loss (>3% of baseline body weight in patients with the above parameters). Given that the results for DHQ and GLP-1 receptor agonists in mice show no significant differences, we can suggest that the flavonoid will demonstrate a similar mean difference in a clinical experiment, which would also be clinically significant. Taking into account the high safety profile of DHQ that was confirmed in cell [110,111] and animal models [112], this flavonoid seems to be a treatment of choice for patients with obesity.

Furthermore, the stereochemistry of the DHQ molecule should be taken into account during the discussion of DHQ pharmacodynamics in obesity. One of the studies enrolled in the systematic review reported the results of the administration of a *cis*-enriched flavonoid substance [113,114]. Surprisingly, the application of this DHQ substance was found to be associated with the most dramatic decrease in rats’ weight at the end of week 4 among all articles that were reviewed [88]. This rather intriguing result might be explained by a higher affinity of the *cis*-diastereomer to biological targets compared to the *trans*-isomer.

### 4.5. Pharmacokinetics and Bioavailability of DHQ

Pharmacokinetics is another aspect that should be taken into account during drug development. After oral administration, DHQ is absorbed in the small intestine. The rest proceeds to the colon, where gut microbiota actively processes it through key reactions such as dehydroxylation, dehydration, and ring-fission. The products of these biotransformations can also be absorbed into the bloodstream. DHQ’s tissue distribution is uneven, with the lowest accumulation observed in the heart and brain, and the highest concentrations found in the kidneys, lungs, and spleen [73]. A major part of flavonoid molecules is bound by blood proteins [88]. Following metabolism in the enterocytes and hepatocytes includes conjugation reactions (glucuronidation, sulfation, and methylation) to facilitate excretion [73,115]. Interestingly, the accumulation of DHQ diastereomers in tissues and the rate of elimination are not equal [116]. The elimination half-life of DHQ is approximately 6.03 ± 1.42 h [115]. The provided description explains the low bioavailability of DHQ and justifies the necessity of applying different techniques to increase the water solubility and permeability of this flavonoid. Turning back to the included studies, in contrast to the data of the previous review [117], the use of solubility modifiers was associated with more pronounced biological effects at lower doses of DHQ. This may be related to the increasing bioavailability due to the rising solubility. For example, a water solution of DHQ with 0.5% sodium carboxymethyl cellulose [89] shows a similar trend, and it may be compared even with an 80 mg/kg dose [92], which had a higher concentration of the flavonoid, but a worse result.

### 4.6. Limitation

This analysis has limitations that should be considered when interpreting the results. Firstly, the heterogeneity is considerable due to variations in diseases studied, animal species used, and impact applied in control groups. However, the target population for weight management with DHQ is also likely to be heterogeneous, encompassing individuals with diverse ages, sexes, and underlying health conditions [118,119]. Therefore, the consistent trend towards weight loss was observed despite the methodological differences between mice and rats. It suggests that DHQ may offer potential benefits for patients with different medical histories in real clinical practice. Additionally, it is important to note that male mice, particularly certain strains like C57BL/6J, are more prone to weight gain compared to rats and humans. This increased susceptibility to weight gain in male mice is attributed to several factors, including their relatively higher levels of testosterone, promoting increased food intake and decreased energy consumption, and a greater tendency to develop visceral obesity [120,121]. Also, additional research is needed to better understand the effect of DHQ on weight loss for adult animals. Moreover, the current experimental model has significant limitations due to fundamental physiological and morphological differences between the experimental animals and humans. For instance, mice and rats have a much higher metabolic rate than humans [122], which can influence how they process and respond to DHQ. Their digestive systems also differ, particularly in terms of gut microbiota composition and function, which can affect the absorption and bioavailability of DHQ. Furthermore, hormonal control of appetite and weight regulation, involving hormones like leptin and ghrelin, varies considerably between rodents and humans [17,123]. Consequently, while these preclinical studies provide valuable initial insights, one can only assume but not be certain that the effect of DHQ on weight loss in humans will be identical to that observed in animal models. This underscores the need to conduct clinical trials in human populations to rigorously confirm the results obtained in these preclinical studies.

### 4.7. Prospects for Further Research

Despite these promising results, some questions remain.

The influence of the DHQ stereochemistry on its anorexigenic effect is unclear. The application of computational methods [124], including molecular docking and molecular dynamics, with respect to the configuration of stereocenters, may shed light on the biological activity of DHQ’s stereoisomers. Nevertheless, the development of chemical approaches that will help to accumulate isomers of DHQ effectively remains necessary to validate the data of in silico methods, using in vitro and in vivo models.

Another short-term goal is to optimize the bioavailability of the flavonoid. It could be achieved by the synthesis of phase modifications [125,126] or innovative dosage forms [127,128]. The enhancement of bioavailability will increase the concentration of DHQ in target organs, which apparently will result in more pronounced biological effects. The progress in this area may be confirmed by a pharmacokinetic experiment in animal models.

The successful solution of chemical and pharmaceutical challenges will form a fundamental basis for translating the results of preclinical experiments into medical practice. If the safety and effectiveness are confirmed in clinical studies through randomized, double-blind, and placebo-controlled trials, the healthcare professionals will obtain a new instrument to control the weight. Taking the case of type 2 diabetes, for example, we may be able to treat patients who require moderate pharmacological intervention. According to data, there are 483 million such patients all over the world [113].

## 5. Conclusions

This systematic review set out to assess the effect of DHQ on the body weight of animal models under different conditions in the context of preclinical studies. Eight articles were included, and the data of 16 rats and 108 mice, treated with different doses of the analyzed flavonoid, in comparison with the 67 animals of the control groups, were summarized. One of the most significant findings to emerge from this study was that the intake of DHQ in the course of one month resulted in a decrease in animals’ weight by 5.24% ± 1.95% (*p* < 0.0001), which is comparable with the preclinical studies of GLP-1 receptor agonists. The second major finding was that the prolongation of DHQ administration for 3 months leads to an increase in body weight loss up to 18.29% ± 1.96% (*p* < 0.0001). Also, the severity of biological effects was associated with the initial animal weight and the pathological condition. To the best of our knowledge, it is the first meta-analysis on the topic. DHQ shows great promise as a weight-loss compound due to its high safety profile and low toxicity. Taken together, our results suggest the need for further research on DHQ as an anorexigenic agent, focusing on the stereochemistry of this flavonoid and its bioavailability optimization.

## Figures and Tables

**Table 1:** Study Inclusion and Exclusion Criteria.

Criteria	Inclusion	Exclusion
Population	Male rats and mice of different lines without conditions causing body weight loss	Use of other species; use of animals with conditions decreasing body weight; female animals or there are no data on the animals’ sex
Intervention(s) or exposure(s)	Intake of DHQ in any formulation and dose	Intake of chemically modified DHQ; intake of mixtures containing several active ingredients except DHQ; doses are not specified in mg/kg or are unconvertible due to insufficient data
Comparator(s) or control(s)	Placebo treatment	Use of any other active ingredients; the condition in the comparison group differs from the treatment group
Outcome measure(s)	Mean ± SD of body weight data (specified or converted in grams) at week 4 or week 12 after the start of DHQ intake	No body weight data are available for these time points; weight is not specified in grams or is presented in units unconvertible to this unit
Study design	Randomized study types	Other study designs
Language	English or Russian	Other languages

**Table 2:** Trial groups’ characteristics.

Reference	Animal Species	Animal Age, Weeks	State	Pathological State Modeling Method	Group Size	
					Treatment Group	Control Group
[88]	Wistar rats	16	Diabetes mellitus type 1	Intraperitoneal injection of 45 mg/kg body weight of fresh 4.5% solution of streptozotocin in cold citrate buffer	8	7
[89]	KK-Ay/TaJcl (KK-Ay/Ta) mice	4	Diabetes mellitus type 2	-	6	7
[87]	C57BL/6J mice	6	Hepatic lipid dysmetabolism mediated by gut microbiota	High-fat diet feeding	8	8
[90]	C57BL/6 mice	8	Hepatic fibrosis and diabetes mellitus type 2	High-fat diet (containing 40% kcal fat) feeding and intraperitoneal injection of 40 mg/kg streptozotocin for 5 consecutive days	6	6
[91]	ICR mice	6–8	Hepatic fibrosis	Intraperitoneal injection of 5 mL/kg body weight of 20% CCl4 peanut oil solution	10	10
					10	
					10	
[92]	Wistar rats	8	Gonadal dysfunction	Oral gavage of 20 μg/kg body weight of bisphenol-A	8	8
[93]	ICR mice	17–19	Hepatic lipid dysmetabolism	High-fat diet (containing 60% kcal fat) feeding and acute ethanol binge	6	6
					6	
					6	
			Healthy	-	6	6
[94]	C57BL/6J mice	5	Obesity	High-fat diet (containing 60% kcal fat) feeding	9	9
					9	

## Data Availability

The original contributions presented in this study are included in the article and Supplementary Materials. Further inquiries can be directed to the corresponding author.

## Supplementary Materials

The following supporting information can be downloaded at: https://www.mdpi.com/article/10.3390/ph18111675/s1, Table S1: Summary of the findings from included studies.

## Abbreviations

The following abbreviations are used in this manuscript:

DHQ	dihydroqurcetin
*ES*	effect size
GLP-1	glucagon-like peptide-1
PRISMA	preferred reporting items for systematic reviews and meta-analyses
RoB	risk of bias
SYRCLE	systematic review centre for laboratory animal experimentation
