# Supplementary material for: Dihydroquercetin in Weight Control: Systematic Review and Meta-Analysis of Preclinical Studies
Source: Pharmaceuticals (Basel). 2025 Nov 5;18(11):1675. doi: 10.3390/ph18111675 (PMC12655469; doi:10.3390/ph18111675)
Supplement: Supplementary file 1 [file pharmaceuticals-18-01675-s001.zip › pharmaceuticals-3945994-supplementary.pdf]

Dihydroquercetin in Weight Control: Systematic Review and Meta-Analysis of Preclinical Studies

Roman P. Terekhov *, Artem A. Svotin, Denis I. Pankov, Maria D. Korochkina, Elizaveta A. Krivosheeva, Elizaveta V. Krivozubova, Ketelina I. Bergel and Irina A. Selivanova

Supplementary Materials

Table S1: Summary of the findings from included studies.

Treatment			Body weight data														Reference
			Weight before treatment, g		Week 4						Week 12						
					DHQ group		Control group				DHQ group		Control group				
Dosage of DHQ (mg/kg)	Administration	Dosage form	DHQ group	Control group	Weight, g	Weight change, %	Weight, g	Weight change, %	Δ, %	*p*-values	Weight, g	Weight change, %	Weight, g	Weight change, %	Δ, %	*p*-values	
50.0	Oral	Suspension in saline solution	283.3	276.7	211.3	-25.41	252.8	- 8.64	-16.77	0.0024451	-	-	-	-	-	-	https://doi.org/10.3390/ijms241511962
30.0	Oral	Suspension in 0.5% sodium carboxymethyl cellulose water solution	28.7	28.4	36.1	+25.78	37.6	+32.39	-6.61	0.4573553	-	-	-	-	-	-	https://doi.org/10.3390/cimb43030092
50.0	Intragastric injection	Suspension in corn oil	23.5	23.0	28.5	+21.28	29.0	+26.09	-4.81	0.4436189	-	-	-	-	-	-	https://doi.org/10.3390/nu14245214
80.0	Oral	-	20.0	20.0	26.0	+30.00	28.0	+40.00	-10.00	0.0002812	36.0	+80.00	46.0	+130.00	-50.00	0.000281262	https://doi.org/10.25073/2588-1140/vnunst.5825
20.0	Intragastric injection	Suspension in saline solution	27.5	27.0	35.0	+27.27	35.0	+29.63	-2.36	-	-	-	-	-	-	-	https://doi.org/10.2147/DDDT.S281369
40.0			29.0	27.0	35.5	+22.41	35.0	+29.63	-7.22	0.3866635	-	-	-	-	-	-	
80.0			29.0	27.0	36.0	+24.14	35.0	+29.63	-5.49	0.0497511	-	-	-	-	-	-	
50.0	Oral	Suspension in 1% methyl cellulose water solution	180.0	170.0	235.0	+30.55	210.0	+23.53	+7.02	0,0000688	-	-	-	-	-	-	https://doi.org/10.1177/09603271221089919
41.2	Oral	Suspension in water	17.5	17.5	25.5	+45.71	24.5	+40.00	+5.71	0.1109223	31.0	+77.14	30.5	+74.29	+2.85	0.634024208	https://doi.org/10.1002/jsfa.11496
73.6			17.0	17.5	24.5	+44.12	24.5	+40.00	+4.12	-	29.5	+73.53	30.5	+74.29	-0.76	-	
1.0	Oral	-	46.0	48.5	44.5	-3.26	52.0	+7.22	-10.48	0.0000001	-	-	-	-	-	-	https://doi.org/10.1039/d0fo02653k
5.0			48.0	48.5	45.0	-6.25	52.0	+7.22	-13.47	-	-	-	-	-	-	-	
25.0			47.5	48.5	45.0	-5.26	52.0	+7.22	-12.48	0.0000002	-	-	-	-	-	-	
25.0			42.5	39.0	41.5	-2.35	41.0	+5.13	-7.48	-	-	-	-	-	-	-	
